# Estimation of salt intake by 24-hour urinary sodium excretion: a cross-sectional study in Yantai, China

**DOI:** 10.1186/1471-2458-14-136

**Published:** 2014-02-08

**Authors:** Jianwei Xu, Maobo Wang, Yuanyin Chen, Baojie Zhen, Junrong Li, Wenbo Luan, Fujiang Ning, Haiyun Liu, Jixiang Ma, Guansheng Ma

**Affiliations:** 1 National Institute For Nutrition and Food Safety, Chinese Center for Disease Control and Prevention, 100050 Beijing, China; 2 Yantai Center for Disease Control and Prevention, 264003 Yantai, China; 3 Yantai Fushan District Center for Disease Control and Prevention, 265500 Yantai, China; 4 Yantai Penglai County Center for Disease Control and Prevention, 265600 Yantai, China; 5 National Center for Chronic and Noncommunicable Disease Control and Prevention, Chinese Center for Disease Control and Prevention, 100050 Beijing, China

**Keywords:** Urinary sodium, Salt intake, Urine

## Abstract

**Background:**

High levels of dietary sodium are associated with raised blood pressure and adverse cardiovascular health. To determine baseline salt intake, we investigated the average dietary salt intake from 24-hour urinary sodium excretion with a small sample of Yantai adults in the Shandong province of China.

**Methods:**

One hundred ninety one adults aged 18–69 years were randomly selected from the Yantai adult population. Blood pressure, anthropometric indices and sodium excretion in a 24-hour urine collection were measured. Consumption of condiments was derived from 3-day weighted records. Completeness of urine collections was verified using creatinine excretion in relation to weight.

**Results:**

The mean Na and K outputs over 24 hours were 201.5 ± 77.7 mmol/day and 46.8 ± 23.2 mmol/day, respectively (corresponding to 11.8 g NaCl and 1.8 g K). Overall, 92.1% of the subjects (96.9% of men and 87.1% of women) had intakes of over 6 g salt (NaCl)/d. The main sources of salt intake from weighed condiments records were from home cooking salt (74.7%) followed by soy sauce (15.0%). Salt intake from condiments and salt excretion were weakly correlated((r = 0.20, *p* = 0.005).A positive linear correlation between salt intake was associated with systolic blood pressure in all adjusted and unadjusted model (r = 0.16, *p* = 0.01). Each 100 mmol/day increase in sodium intake was associated with a 4.0 mmHg increase in systolic blood pressure.

**Conclusion:**

Dietary salt intake in Yantai adults was high. Reducing the intake of table salt and soy sauce used in cooking will be an important strategy to reduce sodium intake among Yantai adults.

## Background

High salt intake is associated with high blood pressure, which, in turn, increases the risk of stroke and cardiac vascular disease [1-4]. Evidence suggests that modest reductions in dietary sodium could substantially reduce cardiovascular events and medical costs, and should be a public health priority [5]. Internationally, calls have been made for dietary sodium reduction to be a major intervention for the prevention and control of non-communicable diseases [6]. In China, hypertension is a major contributor to cardiovascular disease, which accounts for 40% of the deaths [7] and 23% of the health care costs [8]. The China National Nutrition and Health Survey indicated that approximately 80% of Chinese adults exceeded a salt intake of 6 g/day in 2002, the recommendation proposal by the Chinese Nutrition Society [9].

There are several methods for measuring salt intake. These include dietary recall or records from 24 to 96 hours, food frequency questionnaire methods and 24-hour urine collection. Accurate estimation of salt intake is difficult because the amount of salt added during cooking (even in restaurants) and at the table is usually not known. Further, it is difficult to determine the amount of salt remaining on a serving plate and to determine the salt content in food and drinking water [10]. Generally, 24-hour urine collection is considered to be the most reliable method to evaluate salt intake [10-14]. Dietary survey methods tend to underestimate sodium intake [15], while 24-hour urinary sodium excretion is considered the ‘gold standard’ method [16]. However, the 24-hour urinary excretion method does not account for electrolyte loss other than via the kidneys, and therefore will tend to slightly underestimate true sodium intake [14].

In 2011, the China Ministry of Health (MOH) selected Shandong Province as a national pilot area for sodium reduction and the Shandong and MOH Action on Salt reduction and Hypertension (SMASH, 2011–2015) Program was launched to lower sodium intake and to prevent and control hypertension. The SMASH Program is the first provincial-wide, government-led salt reduction program in China; it has adopted a multi-sector collaboration approach and has set its goal to reduce the daily salt intake to less than 10 grams by 2015. In 2011, a cross-sectional baseline survey for the SMASH Program was conducted in the general population aged 18 to 69 years. Yantai in the Shandong Province was selected as a pilot site of the SMASH Program for implementing comprehensive multi-sector strategies to decrease salt intake.The aim of this study is to determine the baseline salt intake among the Yantai adult population. These results will provide information to help develop and implement interventions and to enable evaluation of the effect of the ongoing salt reduction program.

## Methods

### Subjects

The baseline survey was conducted in July 2011. According to the multi-stage cluster sampling of the SMASH program, two counties were selected from 16 counties of Yantai. Using a proportional probability sampling method, three townships and two streets were selected and three villages/neighborhoods were selected from each sampled township/street. In each selected village/neighborhood, 100 subjects were selected by random sampling. A total of 1500 subjects aged 18–69 years were selected. Subjects were invited to an assessment that included administration of a standardized questionnaire, physical examination, and laboratory testing. The study protocol was approved by the Ethical Review Committee of the Chinese Center for Disease Control and Prevention, and participants provided written informed consent.

In addition, we also selected a small subsample from the above sample for a 24-hour urine survey. We randomly selected 5 villages/neighborhoods from 15 sampled townships/streets, and among the selected 100 subjects in selected villages/neighborhoods 42 subjects in each selected village/neighborhood were randomly selected and investigated using 3-day dietary recall assessments and 24-hour urine collections. A total of 207 subjects finished both 3-day dietary recall assessments and the 24-hour urine collections. Sixteen urine collections were excluded because of insufficient recovery. A total of 191 subjects involved in the study were selected for this sub-sample.

### Physical measurement

Physical measurements including height, weight, waist circumference, and blood pressure were conducted following standardized methods. Blood pressure (BP) was measured three times by electronic sphygmomanometer (HEM-7071, Omron Corporation, Japan) and the average of the three measures was calculated as used for analyses. Participants were considered hypertensive if the average systolic BP was ≥140 mmHg or diastolic BP was ≥90 mmHg, or they had a prior diagnosis of hypertension and/or were receiving an anti-hypertensive drug.

### Assessment of salt intake

For three consecutive days (including Thursday, Friday, and Saturday) dietary intake was recorded to assess sources of sodium from condiments (including salt, soy sauce, jam, monosodium and Vinegar). A trained health worker went to the selected individual’s house in the evening, after the family had finished dinner. The health worker recorded the cooking condiments used, purchased and discarded by weight. We measured consumption by weight at the start and end of the assessment period. The sodium and potassium contents in condiments were calculated using reference values from the China Food Composition Table [17].

### 24-hour urine collection and measurements

A single timed 24-hour urine collection was obtained for estimation of electrolyte excretion. Participants were given written and verbal instructions for the 24-hour collection. The first urine of the day was discarded and all urine over the following 24 hours was collected in standard containers that were provided. Total volume of the collection was measured. Urine aliquots were stored at −20°C before being transported frozen to the certified laboratory (ADICON Clinical Laboratory Inc., Jinan, China). In accordance with the standard procedure, urinary sodium and potassium were measured using ion selecting electrode method by Olympus AU 680 autoanalyser (coefficient of variation was 1.5% for sodium and 2.5% for potassium). Creatinine was measured using the picric acid method by Olympus AU640 Analyzer (coefficient of variation was 3.0%).

The 24-hour urine collections were assessed for completeness using creatinine excretion in relation to weight (i.e. the creatinine coefficient = creatinine [mg/day]/body weight [kg]). Creatinine coefficients of 14.4 to 33.6 in men and 10.8 to 25.2 in women were classified as indicating an Acceptable 24-hour urine collection [18]. Daily salt intake was estimated based on calculation of 24-hour urinary sodium excretion on the assumption that all sodium ingested was in the form of sodium chloride.

### Statistical analysis

All statistical analyses were performed using SAS software 9.3 (SAS Inc., Cary, North Carolina, USA). Two-sided *P* < 0.05 was considered statistically significant. Results were expressed as mean ± SD or median and 25th-75th percentiles. Sample characteristics were compared between sexes using a Student’s *t*-test. After salt intake from weighted condiments records was transformed using log, Pearson’s linear correlation was used to detect bivariate associations. Standard linear regression was used to assess associations between sodium and BP, with body mass index (BMI), sex, and age used as covariates.

## Results

### Characteristics of study participants

Demographic data on the participants are given in Table 1. Average urine volume was 1442 (SD 577) mL/d. Average urine creatinine excretion was 9.4 (SD 2.6) mmol/d. Urine creatinine excretion was significantly higher in men (11.0 ± 2.3 mmol/d) than in women (7.6 ± 1.6 mmol/d). Mean potassium excretion was 46.8 (SD 23.2) mmol/d, corresponding with a dietary potassium intake of 1.8 (SD 0.9) g/d in the whole group. The mean sodium:potassium ratio was 4.9 in the whole group and 5.2 and 4.6 in males and females, respectively.

**Table 1:** Characteristics of the study population by sex

	**Men (n 98)**	**Women (n 93)**	**Total (n 191)**	**Statistical value**	**P value**
Age (years)	42.6 ± 13.2	41.5 ± 13.7	42.3 ± 13.5	0.53	0.600
Height (cm)	171.5 ± 6.4	158.6 ± 6.5	165.1 ± 9.1	13.93	<0.0001
Weight (kg)	73.8 ± 12.7	62.5 ± 11.8	68.4 ± 13.4	6.38	<0.0001
BMI (kg/m^2^)	25.1 ± 4.1	24.9 ± 4.5	25.0 ± 4.3	0.33	0.743
Waist circumference (cm)	86.7 ± 11.5	80.1 ± 11.3	83.6 ± 12.0	3.99	<0.0001
Blood pressure					
Systolic (mmHg)	128.0 ± 19.9	118.0 ± 19.4	123.5 ± 20.3	3.54	0.001
Diastolic (mmHg)	82.8 ± 12.0	77.1 ± 11.6	80.2 ± 12.1	3.32	0.001
Urinary excretion					
Volume (ml/d)	1509 ± 571	1370 ± 578	1442 ± 577	1.68	0.094
Creatinine (mmol/d)	11.0 ± 2.3	7.6 ± 1.6	9.4 ± 2.6	11.34	<0.0001
Sodium (mmol/d)	218.3 ± 81.4	183.8 ± 69.8	201.5 ± 77.7	3.14	0.002
Potassium (mmol/d)	45.9 ± 17.9	47.7 ± 27.7	46.8 ± 23.2	−0.53	0.594
Sodium-to-potassium ratio	5.2 ± 2.2	4.6 ± 2.2	4.9 ± 2.2	1.97	0.05
Salt intake (g/d)	12.8 ± 4.8	10.8 ± 4.1	11.8 ± 4.5	3.14	0.002

### Sodium and salt intake in adults

**Figure 1:**
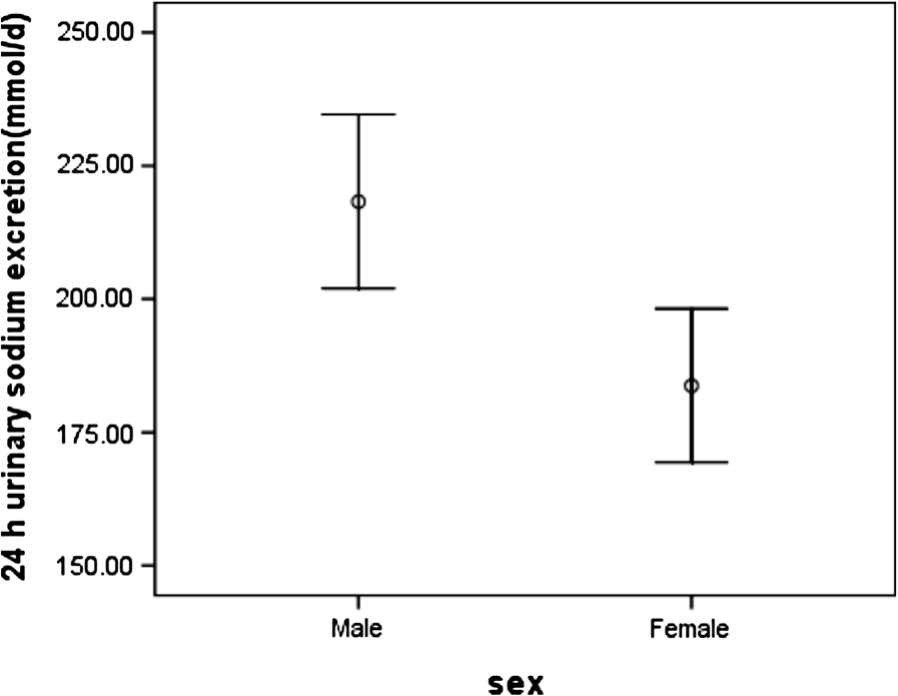
The estimate of mean value of 24 h urinary sodium excretion by gender.

Urinary sodium excretion in the whole group was 201.5 (SD 77.7 mmol/d), which corresponds to 11.8 (SD 4.5) g/d NaCl. Urinary sodium excretion was significantly higher in men (218.3 ± 81.4 mmol/d) than in women (183.8 ± 69.8 mmol/d; *p* = 0.002, Figure 1), corresponding to 12.8 (SD 4.8) g/d and 10.8 (SD 4.1) g/d NaCl, respectively. Overall, 92.1% of the participants (96.9% of men and 87.1% of women) had intakes of over 6 g salt (NaCl)/d (102 mmol of Na/d) (recommended Chinese maximum ).

### Salt intake from weighed condiments records

For the whole population, the median salt intake from weighed condiment records was 10.1 g/d. The main source of salt was from home cooking salt (74.7%), followed by soy sauce (15.0%), and other condiments (10.3%). Salt intake from weighed condiments was 10.1 g/d compared with 11.8 (SD4.5) g/d from urinary Na excretion (*p* = 0.575). Salt intake from condiments and salt excretion were weakly correlated (r = 0.20, *p* = 0.005) (Table 2).

**Table 2:** Correlation between salt intake from condiments and salt excretion

**Condiments usage (g/d)**	**Median**	**25th–75th percentiles**	**%**	**Correlation coefficients**	**P value**
Salt	7.3	5.7–11.3	74.7	0.19	0.017
Soysauce	1.4	0.6–2.4	15.0	0.065	0.389
Jam	0.2	0–0.7	4.7	0.129	0.202
Monosodium	0.4	0.2–0.9	5.2	0.322	0.001
Vinegar	0.03	0–0.07	0.4	0.154	0.03
Total	10.1	7.6–15.6	100	0.20	0.005

### Urinary sodium and blood pressure

Systolic BP (r = 0.25, *p* = 0.0006) and diastolic BP (r = 0.22, *p* = 0.0020) were positively and significantly correlated with 24-hour urinary Na excretion. Adjusting for sex, age, and BMI, linear regression analyses found that sodium excretion was positively associated with systolic BP in all adjusted models. After adjusting for sex, age, and BMI, no significant association between urinary sodium and diastolic BP was found (Table 3).

**Table 3:** Relationship between blood pressure and urinary sodium

**Variable**	**β**	**SE**	**Standardized β**	**P Value**
Systolic blood pressure				
Unadjusted	0.06441	0.01835	0.24740	0.0006
Model 1*	0.05091	0.01668	0.19553	0.0026
Model 2*	0.04012	0.01617	0.15794	0.0118
Diastolic blood pressure				
Unadjusted	0.03460	0.01102	0.22277	0.0020
Model 1*	0.02724	0.01071	0.17536	0.0118
Model 2*	0.01925	0.01008	0.12394	0.0576

*Model 1: adjusted for age and sex; Model2: adjusted for age, sex and body mass index.

## Discussion

We found the average urinary sodium excretion among adults in Yantai was 201.5 (SD 77.7) mmol/d (11.8 g salt/d).This level is double the recommended intake of 5 g salt/d issued by the WHO in 2012[19] and the China Nutrition Recommendations[20]. Thus, salt intake among Yantai adults is very high.

For China, only limited data on sodium intake assessed using 24-hour urine collection is available. Liu *et al*. reported urinary sodium excretion was 188.4 (SD 86.0) mmol/d in a population of 48–56-year-olds in China [21]. Most studies find the intake and excretion of Na higher among men, compared to women, perhaps because of their overall higher food intake and differences in dietary habits [10,16,22].

In China, salt intake is high mainly because of the tradition of adding excessive salt to foods (both during cooking and at the table) and the high consumption of soy sauce [10,23,24]. A recent study suggested that most dietary sodium (76%) in China was from salt added in home cooking [25]. The results of the present study also show that the main source of salt was from home cooking salt (74.7%) and soy sauce (15.0%). A weak correlation between salt intake from condiments and salt excretion was obtained. By comparison, most of the salt in western countries, in fact 75–80%, is derived from processed foods [10].

Linear regression models clearly demonstrated a positive linear association between urinary sodium and systolic BP in this study. Accordingly, we found a reduction in sodium intake of 100 mmol/d (6 g salt/day) was associated with a 4.0 mmHg reduction in systolic BP. Our findings provide supporting evidence that the current high intake of sodium is related to higher BP among the population. This finding is consistent with the Intersalt international cross-population study [26] and the SALTURK study [27], which estimated that a 100 mmol/day lower sodium intake was associated with 2.2 mmHg and 5.8 mmHg lower systolic BP, respectively. An Australian study found that a reduction in sodium intake of 100 mmol/d was associated with a 2.3 mmHg reduction in systolic BP [28]. Importantly, a population-wide fall in systolic BP of 2 mmHg has been predicted to lower stroke mortality by 10% and ischemic heart disease and other vascular diseases by 7% [29].

A major strength of our study is that we used the ‘gold standard’ 24-hour urine collection to estimate sodium intake. Completeness of the urine sample was ascertained through urinary creatinine excretion. However, our study has some limitations, including a small sample size because of the pilot nature of the study, which limits the precision to estimate the effect strength of salt intake on BP and Additionally, a single measure was not able to assess the impact that day-to-day variability of sodium intake will have on urinary sodium excretion. For assessment sources of salt intake, this study only deals with added salt during home cooking and not with salt in the total diet. For the linear regression analyses, some potential confounding factors such as physical activity, total energy intake, alcohol consumption, and family history of hypertension, have not been adjusted.

## Conclusions

The present results show that dietary salt intake in Yantai adults was high. Higher salt intake was associated with higher systolic BP. Effective reduction of salt consumption requires specific targeting of those condiments in home cooking that contribute most to salt intake, including cooking salt and soy sauce.

## Competing interests

The authors declare that they have no competing interest.

## Authors’ contributions

MW, JM and GM conceived and designed the study. MW, YC, BZ, JL, WL, FN and HL performed the experiments. JX and MW conducted the data analyses and drafted the manuscript. All authors read and approved the final manuscript.

## Pre-publication history

The pre-publication history for this paper can be accessed here:

http://www.biomedcentral.com/1471-2458/14/136/prepub
